# Substances in the Nose

**Published:** 1884-11

**Authors:** 


### SUBSTANCES IN THE NOSE.

A KERNEL of corn, or a pea, or other small substance, gets into a child's nose, and the problem is to remove it. A recent medical journal proposes to administer an emetic, and then, at the moment vomiting is about to begin, press a handkerchief or napkin firmly over the mouth. The vomited matter must then pass through the nose, and the force of its current will wash away the intruding substance. We have not seen this plan tried and cannot vouch for its efficacy or safety. It seems to us that the volume of the discharge from the stomach would be too great to pass through the nose, even if both nostrils were unobstructed. If so, regurgitation into the æsophagus and larynx, with consequent strangling, would result.

We would suggest the following method as less objectionable and quite as effectual in accomplishing the object desired. Inject into the free nostril with a common syringe a stream of tepid water. The sides of the nostril should be closed around the point of the syringe to prevent a reflux of the water. Use a moderate degree of force and the stream will mske a circuit through the posterior nasal cavity and pass out of the opposite nostril, dislodging the foreign substance.

A more simple method is this. The patient takes a "deep breath," then closes the mouth and the free nostril and forces his breath through the obstructed nostril. If the child is old enough to do it perfectly he may blow out the corn or pea, or whatever has found a lodgment in the nasal passage.

